# Formation Mechanism and Control Method of Residual Stress Profile by Laser Shock Peening in Thin Titanium Alloy Component

**DOI:** 10.3390/ma14081878

**Published:** 2021-04-09

**Authors:** Xiangfan Nie, Yuyuan Tang, Feifan Zhao, Li Yan, Haonian Wu, Chen Wei, Weifeng He

**Affiliations:** 1 Science and Technology on Plasma Dynamics Laboratory, Air Force Engineering University, Xi’an 710038, Shaanxi, China; skingkgd@163.com (X.N.); 565921690@163.com (Y.T.); whyiong@163.com (C.W.); hehe_coco@163.com (W.H.); 2 School of Mechanical and Power Engineering, East China University of Science and Technology, Shanghai 200237, China; 3 School of Aeronautics, Chongqing Jiaotong University, Chongqing 400074, China; 622170990005@mails.cqjtu.edu.com

**Keywords:** laser shock peening, titanium alloy sheet, residual stress-strain profile, shock wave propagation, material dynamic response

## Abstract

In the laser shock peening process of titanium alloy thin blades, a shock wave will be repeatedly reflected and coupled in the blades, resulting in the failure of the formation of a gradient residual compressive stress layer, which is the key to improve fatigue performance and resist foreign object impact. This paper takes TC17 titanium alloy sheet as the research object to reveal the influence mechanism on residual stress-strain profile of shock wave reflection-coupling by shock wave propagation and key position dynamic response. Based on the result of influence mechanism, two wave transmission methods are proposed to regulate shock wave in order to reduce the intensity of shock wave reflection. The analysis shows that the high strength stress be formed when the shock wave is reflected and coupled in the sheet, which causes the re-plastic deformation and the decrease of transverse plastic strain. This eventually leads to residual tensile stress up to 410 MPa being formed within a 0.5 mm radial direction and 0.3 mm deep of the spot range. The use of “soft” and “hard” wave-transmitting layers greatly reduces the shock wave reflection intensity, and under the condition of the “hard” wave-transmitting layer, a better impedance matching is achieved, resulting in a residual compressive stress of about 400 MPa.

## 1. Introduction

Titanium alloy is widely used as a structure material of aero-engine fan and compressor blades, which are subject to high-cycle fatigue cracks and fractures due to rotating centrifugal load, air-excited vibration force, and foreign object impact [1]. Laser shock peening (LSP), as a surface anti-fatigue strengthening technology, uses short-pulse and high-power laser induced plasma shock wave to cause plastic deformation on material surface and higher residual compressive stress, thereby inhibiting the initiation of fatigue cracks, reducing the rate of crack growth, and improving the fatigue strength and life of components [2]. The compressive residual stresses induced by LSP can extend even up to several millimeters deep into the material, much deeper in respect to other conventional techniques [3]. Therefore, LSP has been used as an important technical means to enhance the fatigue resistance and life extension of aircraft components [4].

Residual compressive stress is the main factor for LSP to improve the fatigue resistance of materials. Therefore, the residual stress profile is an important basis for optimization and evaluation of the strengthening process. The surface layer of the blade after LSP has mostly formed residual compressive stress significantly improves the fatigue life [2,5,6]. As the thrust-to-weight ratio of aero engines increases, the design of titanium alloy blades is developing towards integration and weight reduction, and blades are being designed thinner and thinner [7]. However, the LSP results of thin components are not ideal: K. Ding [8] performed LSP simulation of thin sections of TC4 with different thickness (1~3 mm), and Boyu Sun [9] performed LSP experiments of TC17 samples with different thicknesses (1~5 mm). These studies found that as the thickness of the sample decreases, the residual compressive stress decreases significantly. An experiment for laser peening Ti17 sheets was conducted by Cellard [10]. For thin specimens, residual tensile stress was found. Researchers have also conducted certain studies on the reasons for this phenomenon. M. Burak [11] used different laser peening parameters to experiment on Al2024-T351 plate. Their study shows that laser peening can introduce compressive strains in thin plates. Some of the compressive strains can be relaxed, however, due to distortion. Meanwhile, they point out complex interactions between laser-induced shock wave and the plate boundaries make it difficult to assess the residual stress formation mechanisms in thin plates. Kristina Langer [4] analyzed the laser peening of aluminum alloy sheet through finite element method and found that the reflected stress wave limits the formation of the strain profile, which in turn affects the magnitude of the residual stress; G. Ivetic [12] performed an LSP simulation on Al2024 and found that inappropriate laser peening parameters, such as higher plasma pressure and shorter pulse duration, did not produce any significant plastic deformation in the treated sheets.

**Figure 1:**
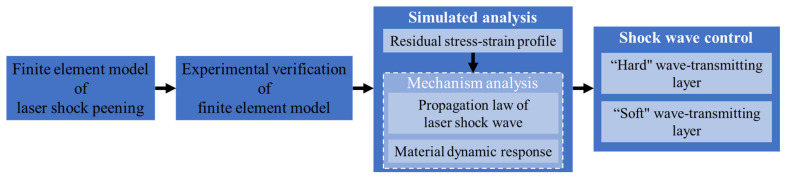
Flowchart of research design.

The above research results show that thin components are peened by laser, and effective residual compressive stress cannot be formed; even residual tensile stress is formed. Although related studies have shown that the root cause is the repeated reflection and coupling of shock wave in the material, most of the existing studies focus on the residual stress profile in order to select appropriate shock laser peening parameters by simulations or experiments. The influence mechanism of shock wave reflection-coupling process on the residual stress-strain profile is unclear. Therefore, as shown in Figure 1, a finite element laser shock model verified by experiments is established. Through the analysis of the simulation results, this paper focuses on the study of the reflection-coupling laws of laser shock wave in TC17 sheet and the dynamic mechanical response characteristics of materials to reveal the mechanism of shock wave reflection-coupling on the residual stress profile. According to the stress wave transmission theory, two wave transmission methods are proposed to realize the active control of shock wave, and the X-ray Diffraction (XRD) results of single-sided laser peening TC17 titanium alloy blades under the two wave transmission methods are analyzed.

## 2. Laser Shock Peening Model

### 2.1. Titanium Alloy Material Model

The Johnson-Cook (J-C) material model [13,14] was adopted, which is widely used under high strain rate conditions such as high-speed impact and explosion impact. Taking into account the absorption protection layer and water confinement layer on the surface of samples, the thermal effect is ignored [15,16]. The model is rewritten as Equation (1):

(1) $$\sigma_{y}=\left(A+B\varepsilon^{n}\right)\left(1+Cln\left(\dot{\varepsilon }/{\dot{\varepsilon }}_{0}\right)\right)$$

where A is the yield strength of the material, B is the work hardening modulus, *n* is the hardening coefficient, C reflects the strain rate hardening effect of the material, ε is the plastic strain, and $\dot{\varepsilon }/{\dot{\varepsilon }}_{0}$ is the dimensionless plastic strain rate.

As the strain rate of the material in SHPB (split Hopkinson pressure bar) is lower (10^4^/s) than that in the laser peening process (10^6^/s), this study uses the J-C parameters identified by our group based on Levenberg-Marquardt algorithm, as detailed in reference [17] (Table 1).

### 2.2. Finite Element Model

The plastic deformation layer of titanium alloy by laser shock can reach more than 1 mm [10]; therefore, plastic wave will be reflected in a thin component (1 mm thick). In order to reveal the reflection-coupling law of laser shock wave and the difference of the influence of shock wave reflection and non-reflection on the dynamic response of materials, two models are established in this study.

**Figure 2:**
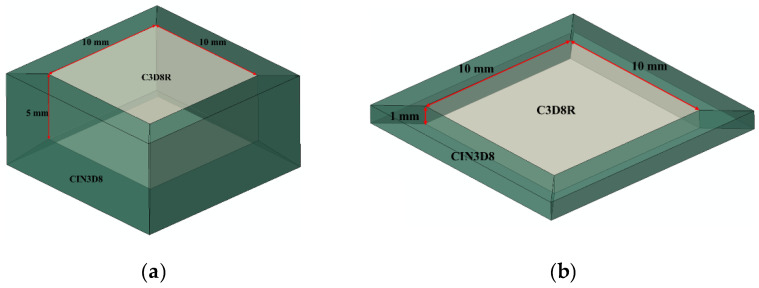
3D Model. (**a**) Infinite plate; (**b**) 1 mm sheet.

One is an infinitely thick model: a 10 mm × 10 mm × 5 mm cube, infinite elements CIN3D8 are set as a non-reflective boundary condition to eliminate the affection of shock wave reflection, the central area is finite elements C3D8R, as shown in Figure 2a. The other is a sheet: the model is a 10 mm × 10 mm × 1 mm cube. The infinite elements CIN3D8 are set at the circumferential of the model to eliminate the affection of the shock wave circumferential reflection on the axial shock wave, the central area is finite elements C3D8R. This model is used to study the dynamic stress in the sheet to characterize the reflection process of the shock wave, as shown in Figure 2b. Mesh size has been set to 0.1 mm to enable correct shock wave propagation through the whole model, without any loss of information. Mesh sensitivity has been checked.

**Figure 3:**
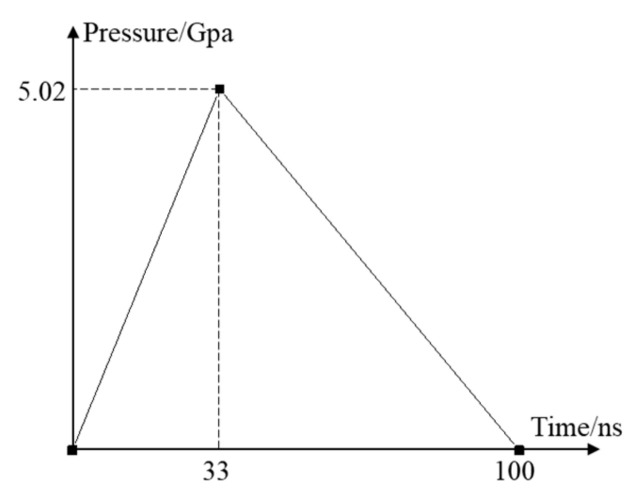
Pressure-time curve.

**Figure 4:**
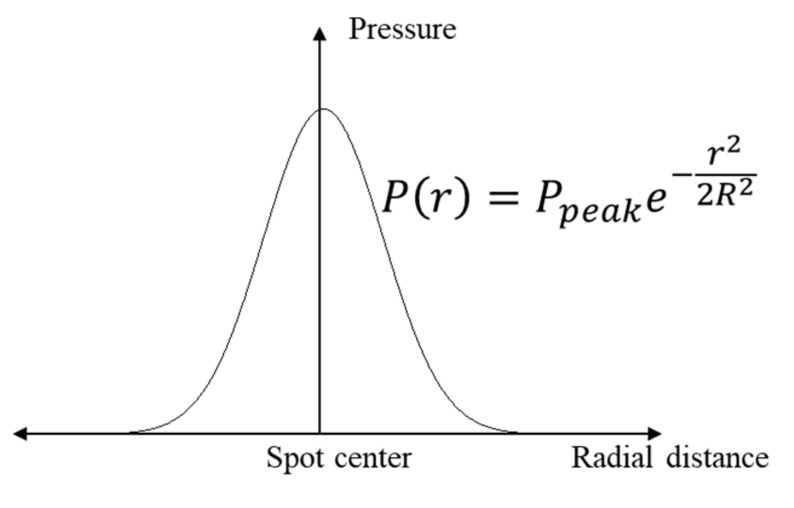
Pressure-space curve.

Use Nd: YAG pulsed laser, power density 4.24 GW/cm^2^, corresponding to specific laser parameters: wavelength 1064 nm, pulse width 20 ns, spot diameter 3 mm, laser energy 6 J. Black tape was pasted on the impact surface and water was used as a constraining layer. Calculated by the Fabbro [18] model (Equation (2)), the peak pressure of the laser shock wave is 5.02 Gpa. The simplified pressure-time curve was used (Figure 3). The pressure is Gauss profile along the radial direction of the spot [19] (Figure 4), and Equation (3) is used to complete the definition of the laser shock load.

(2) $$P_{peak}\left(\mathrm{GPa}\right)=0.01{\left(\frac{\xi }{2\xi +3}\right)}^{0.5}Z^{0.5}\left(g\cdot {\mathrm{cm}}^{-2}\cdot s^{-1}\right)I^{0.5}\left(\mathrm{GW}\cdot {\mathrm{cm}}^{-2}\right)$$

In the equation, ξ is a constant 0.25, Z is the reduced shock impedance $0.832\times 10^{6}g\cdot {\mathrm{cm}}^{-2}\cdot s^{-1}$, and I is the laser power density 4.24 GW/cm^2^.

(3) $$P\left(r,t\right)=P_{peak}P\left(t\right)\exp\left(-\frac{r^{2}}{2R^{2}}\right)$$

where $P\left(r,t\right)$ is the shock wave pressure at a certain point and moment, $P_{peak}$ is the peak pressure of the shock wave, $P\left(t\right)$ is the time amplitude curve, R is the spot radius, and r is the distance from a certain point to the spot center.

In the LSP simulation, numerical calculation using ABAQUS can be classified into two stages: explicit dynamics analysis is performed using the ABAQUS/Explicit to determine the steady-state solutions and calculate the short duration shock wave until the saturation of plastic deformation occurs in the target. The deformed body with all transient stress and strain states is imported into the ABAQUS/Standard to finally determine residual stress fields at static equilibrium.

### 2.3. Model Validation

**Figure 5:**
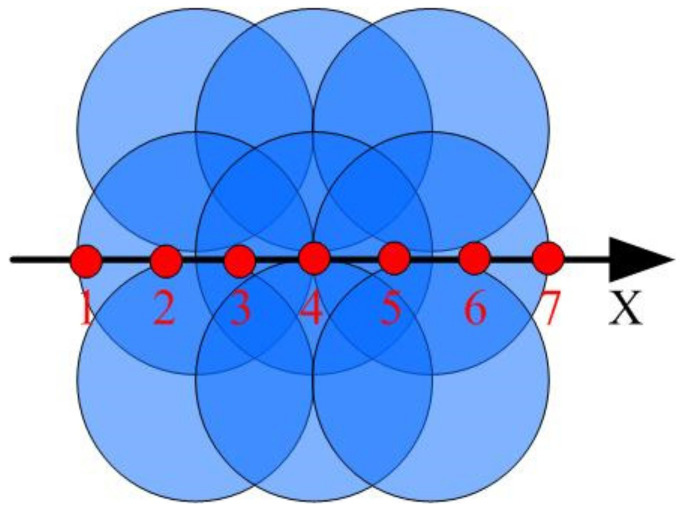
Residual stress testing plan.

Three sets of LSP are carried out on the TC17 samples. The XRD is used to test the stress distribution in the radial directions of the sample surface after impact. The specific test plan is shown in Figure 5: on the surface of the treated area, a total of 7 points were tested every 1 mm along the X axis. The residual stress was determined using the fixed inclination method by a Photo-LXRDX-ray diffraction device from Proto Inc. (Oldcastle, ON, Canada). The Cu-Kα characteristic curve, the wavelength of 1.5418 Å, the diffraction crystal plane Ti(213), tube voltage 35 KV, tube current 30 mA, quasi-diameter 1 mm.

**Figure 6:**
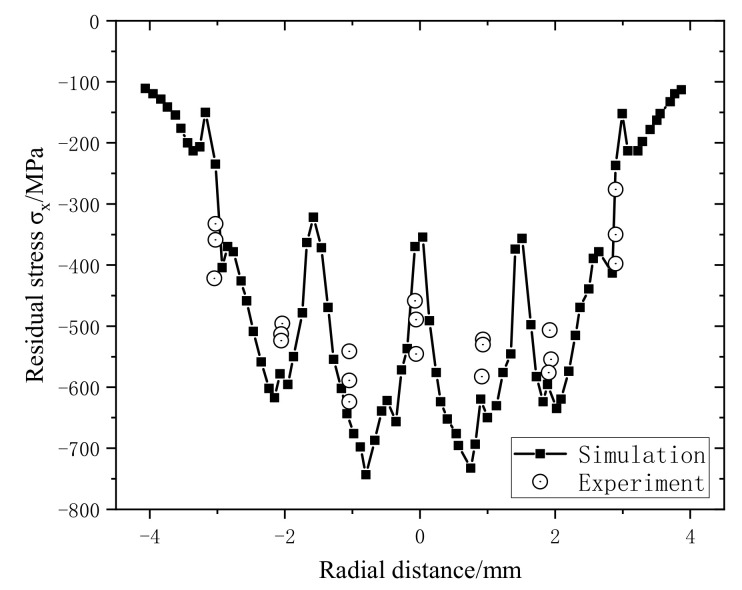
Comparison of residual stress results.

Figure 6 shows the comparison between the XRD test results and the numerical simulation results. Along the X-axis, residual compressive stress of 500~600 MPa is formed in the overlapping area of the spot, but the distribution is not perfectly symmetrical, mainly because the overlapping sequence of the spot affects the stress profile. The numerical simulation model is in good agreement with the experimental results and can be used for subsequent analysis and research.

## 3. Results and Analysis

### 3.1. Residual Stress-Strain Profile

**Figure 7:**
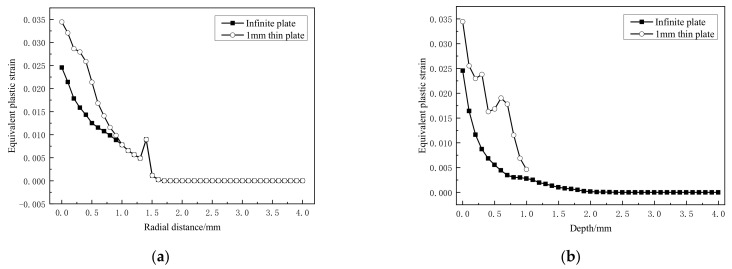
Equivalent plastic strain. (**a**) Radial direction; (**b**) depth direction.

**Figure 8:**
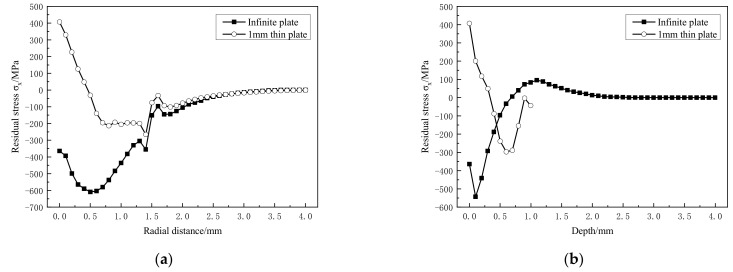
Residual stress. (**a**) Radial direction; (**b**) depth direction.

After single point laser peening, the equivalent plastic strain curve and residual stress curve are shown in Figure 7 and Figure 8, respectively.

Compared with the infinite thickness plate, it is found from Figure 7a that the equivalent plastic strain in the radial direction of the spot 1.0 mm increases significantly. A significant increase also shows on that in the depth direction (Figure 7b). It shows that the strong reflection of the shock wave leads to re-plastic deformation, which affects the entire residual strain profile.

The re-plastic deformation of the material causes the difference in the strain profile, and also leads to the change of the final residual stress profile. Compared with the infinite thickness plate, the sheet formed a tensile residual stress up to 410 MPa in the radial 0.5 mm of the spot after laser peening. Although the residual compressive stress is formed outside 0.5 mm in the radial direction, the compressive stress value is very small compared to the infinite thickness model, only 200 MPa (Figure 8a); the residual tensile stress is also formed within 0.3 mm in the depth direction (Figure 8b). No effective residual compressive stress layer is formed on the material surface or in the depth direction.

### 3.2. Analysis on the Formation Mechanism of Residual Stress-Strain Profile

#### 3.2.1. Propagation Law of Laser Shock Wave in Sheet

**Figure 9:**
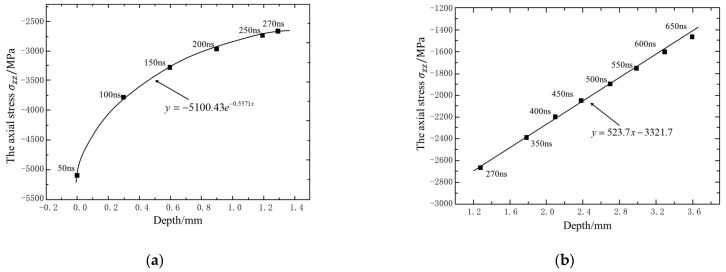
Pressure attenuation curve. (**a**) Elastic-plastic waves; (**b**) plastic waves.

Figure 9 shows the maximum wavefront pressure attenuation curve of the elastic wave and plastic wave in the infinite thickness model; 50~270 ns, the peak value of shock wave, is higher than the dynamic elastic limit of the material, the propagation stage of elastoplastic wave. The peak pressure of longitudinal wave in the depth direction decays exponentially [20] with $P\left(\mathrm{MPa}\right)=-5100e^{-0.5571x}$, and the plastic influence layer reaches more than 1.3 mm (Figure 9a); after 270 ns, the elastic wave propagation stage, the peak pressure of longitudinal wave decays linearly with $P\left(\mathrm{MPa}\right)=523.7x-3321.7$ (Figure 9b). Compared with the elastoplastic wave propagation stage, the attenuation rate of the peak pressure of the longitudinal wave in the elastic wave propagation stage is greatly reduced. This is mainly because there is no plastic deformation at this stage, which slows conversion and dissipation of kinetic energy.

Due to the plastic influence layer is more than 1 mm (Figure 9a), the shock wave will be strongly reflected on the back, which affects the distribution of residual compressive stress. The following is a numerical simulation of single laser peening for a sheet to analyze the shock wave reflection law and its influence mechanism on the residual stress profile.

**Figure 10:**
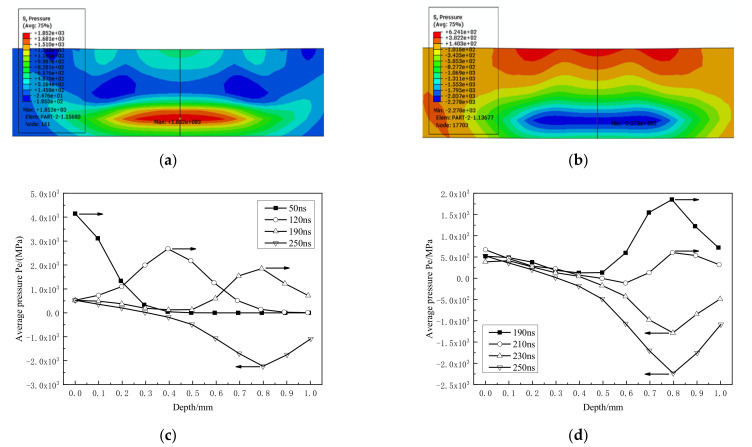
Reflection of the first stress wave on the back. (**a**,**b**) 190 ns, 250 ns stress contour; (**c**,**d**) propagation and reflection process.

Figure 10 shows the first reflection process of the shock wave on the back side. As shown in Figure 10c, the peak pressure of the shock wave before reflection attenuates from 4147.6 MPa at 50 ns to 1851.6 MPa at 190 ns, but the peak pressure becomes −2235.2 MPa after reflection, indicating that the incident compression wave forms a tensile wave after reflection. The increase in pressure value is due to the coupling between the reflected tensile wave and the tensile unloaded wave after the incident compression wave [21]. It can be seen from Figure 10d that the reflected shock wave is gradually formed within 190~250 ns, and the peak pressure of the reflected tensile wave is located at the sub-back (0.8 mm).

**Figure 11:**
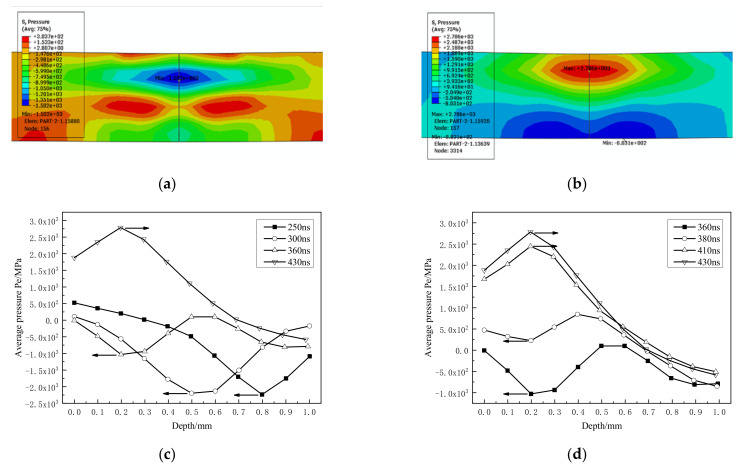
Reflection of the second stress wave on the front. (**a**,**b**) 360 ns, 430 ns stress contour; (**c**,**d**) propagation and reflection process.

The second reflection completes on the impact surface, 360~430 ns (Figure 11), the property of the shock wave changes from tensile wave to compression wave, and the pressure value also increases significantly, reaching 2786.1 MPa at 430 ns. The compression wave peak pressure is gradually formed on the subsurface (0.2 mm deep) during the reflection process.

**Figure 12:**
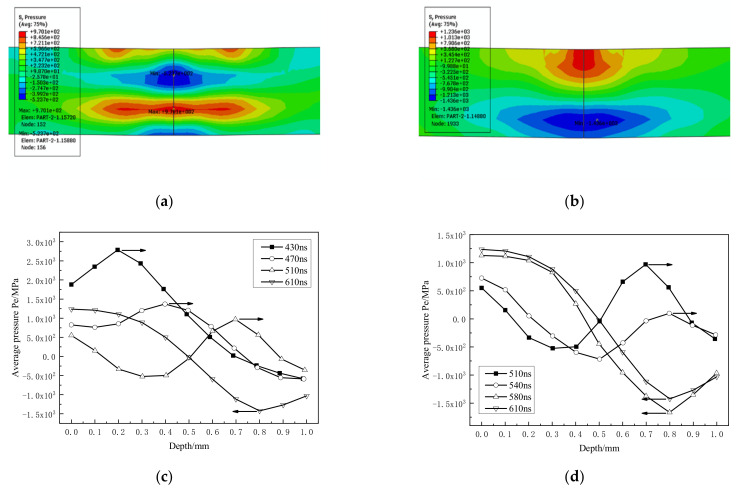
Reflection of the third stress wave on the back. (**a**,**b**) 510 ns, 610 ns stress contour; (**c**,**d**) propagation and reflection process.

When the shock wave propagates to the back face again (Figure 12), the pressure value attenuates from 2786.1 MPa at 430 ns to 970.1 MPa at 510 ns, and the attenuation degree reaches 65%. After the reflection is completed, the average pressure becomes −1423.8 MPa. It also shows that when the stress wave properties change, the pressure value also increases.

#### 3.2.2. Material Dynamic Response

Through the above research and comparison with the infinite thickness model, the strong reflection and coupling laws of shock wave in the sheet have been found. In order to further reveal the impact mechanism of shock wave reflection on the residual stress-strain profile, an analysis on the dynamic mechanical response of materials at the key positions of shock wave propagation and residual stress profile characteristics.

**Figure 13:**
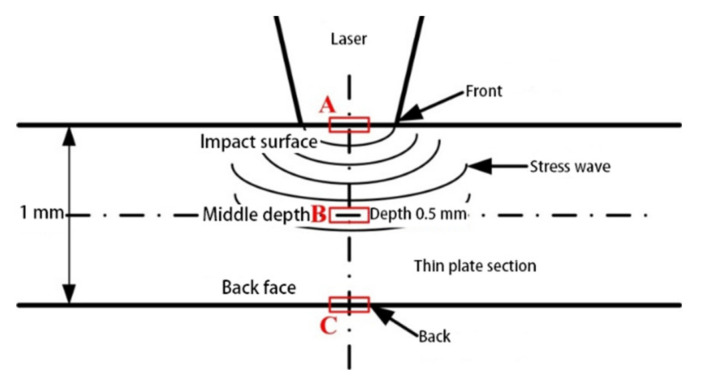
Three key position of dynamic response.

As shown in Figure 13, three key positions in the depth direction of the sheet are selected: impact surface (A), middle depth (B), and back face (C).

**Figure 14:**
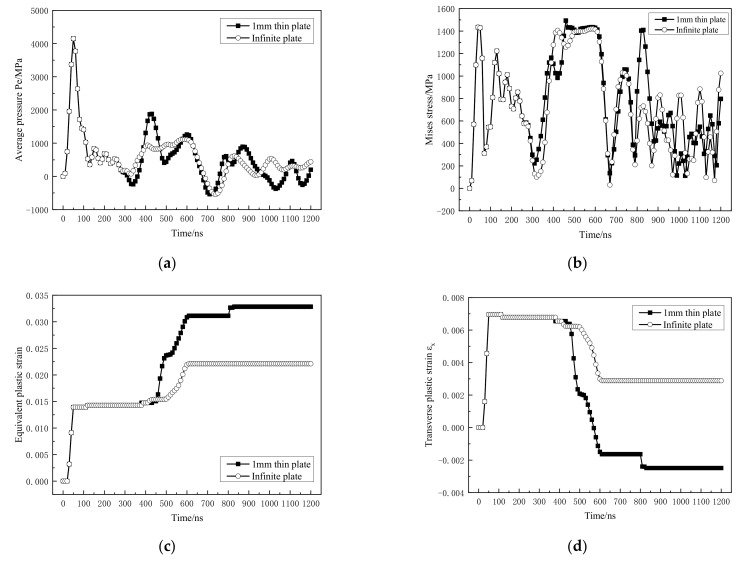
Dynamic mechanical response curve at A position. (**a**) Average pressure; (**b**) Mises stress; (**c**) equivalent plastic strain; (**d**) transverse plastic strain.

Figure 14 is the dynamic mechanical response curve of the material at A. Compared with the infinite-thickness model, in Figure 14(a), starting from 300 ns, the material at A is subjected to alternating tension-compression stress waves. From the analysis of Section 3.2.1, the first and second reflection of the shock wave on the impact surface results in the increase of the peak Mises stress of the material at 400~600 ns and 800 ns at A (Figure 14a). This leads to an increase in the plastic deformation of the material within 400~600 ns and plastic deformation at 800 ns (Figure 14c), which leads to an increase in the equivalent plastic strain of the final impact surface in Section 3.1. Moreover, both shock wave reflections cause the transverse plastic strain to decrease, and even become negative (Figure 14d), indicating that the material at the center of the spot is in a state of transverse compression, and the residual tensile stress shown in Figure 8 is formed.

**Figure 15:**
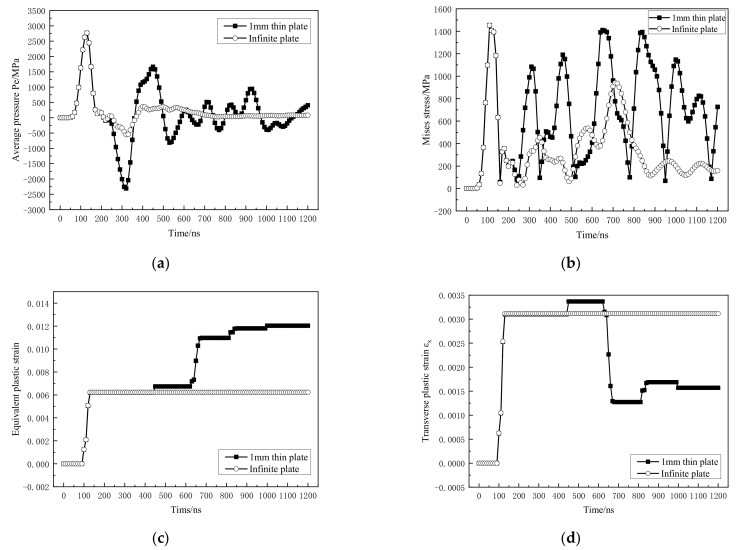
Dynamic mechanical response curve at B position. (**a**) Average pressure; (**b**) Mises stress; (**c**) equivalent plastic strain; (**d**) transverse plastic strain.

Figure 15 is the dynamic mechanical response curve of the material at the intermediate depth B. From the propagation of the shock wave in Section 3.2.1. it is known that due to the continuous reflection of the shock wave on the impact surface and back, the material at B is subjected to the alternating action of tension-compression stress (Figure 15a). This causes the Mises stress to increase significantly and shows a “multimodal” form (Figure 15b). The alternating stress also causes the plastic deformation to change accordingly. It can be seen in Figure 15c that the intermediate material undergoes four plastic deformations under repeated stress. In Figure 15d, the four plastic deformations make the material’s transverse plastic strain alternately change, that is, the material is repeatedly undergoing tensile and compressive plastic deformation. With the attenuation and reflection of the shock wave, the degree of plastic deformation caused by the shock wave also gradually decreases.

**Figure 16:**
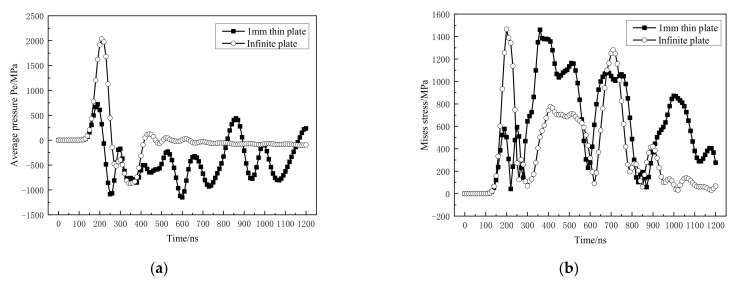

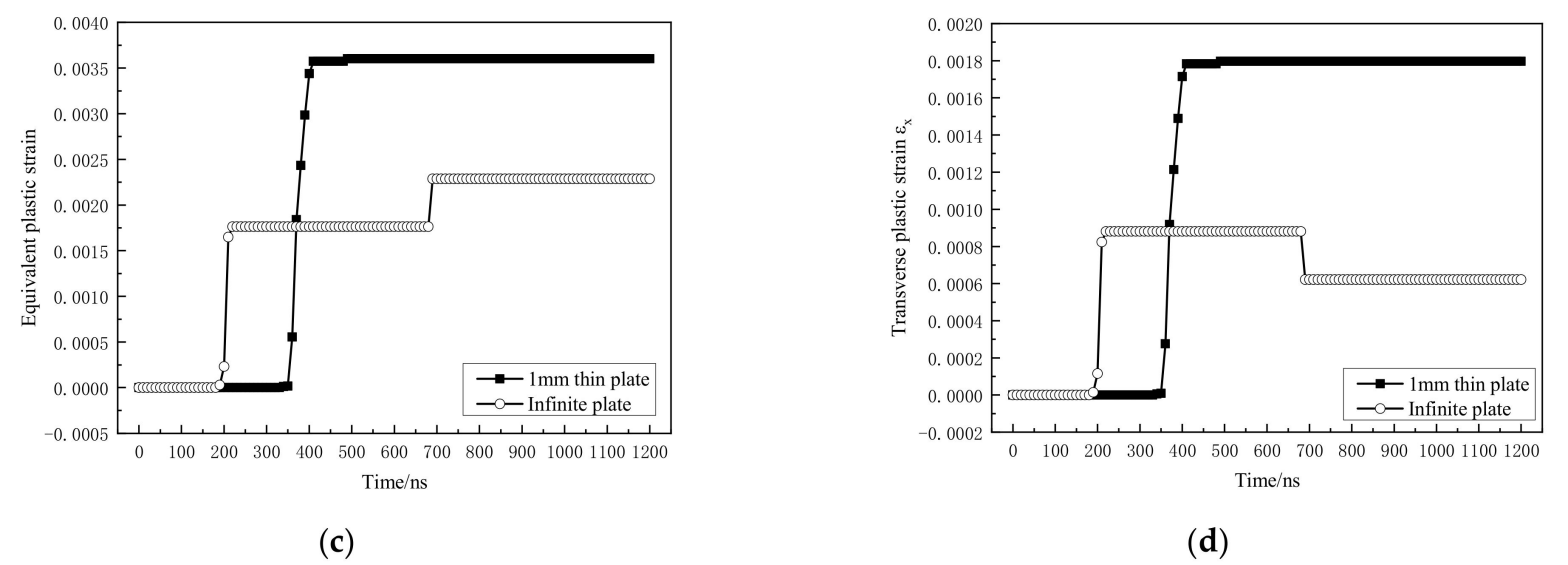
Dynamic mechanical response curve at C position. (**a**) Average pressure; (**b**) Mises stress; (**c**) equivalent plastic strain; (**d**) transverse plastic strain.

Figure 16 is the dynamic mechanical response curve of the material at the back C. As shown in Figure 16a, since the reflected tensile wave is coupled with the incident compression wave during the reflection process, the shock wave pressure is lower than that under the condition of an infinite plate. As shown in Figure 16b, the Mises stress has increased significantly within 300~500 ns. Corresponding to Figure 16c,d, due to the sharp increase in the Mises equivalent stress of the material within 300~500 ns, the material undergoes severe plastic deformation at 360 ns and the transverse plastic strain increase.

### 3.3. Residual Stress-Strain Profile Control Method and Experimental Verification

When single-sided LSP is performed on the thin components, it is necessary to actively control the laser shock wave to reduce the shock wave reflection intensity to ensure that an effective residual compressive stress layer can be formed [22]. Based on the theory of stress wave reflection and transmission, this paper proposes a single-sided LSP method based on the wave-transmitting layer.

Adding a wave-transmitting material that matches the acoustic impedance of the thin blade on the back of the thin blade will effectively eliminate the shock wave reflection and make the shock wave be transmitted directly. The design and verification of the wave-transmitting layer structure of two different materials will be carried out below.

**Figure 17:**
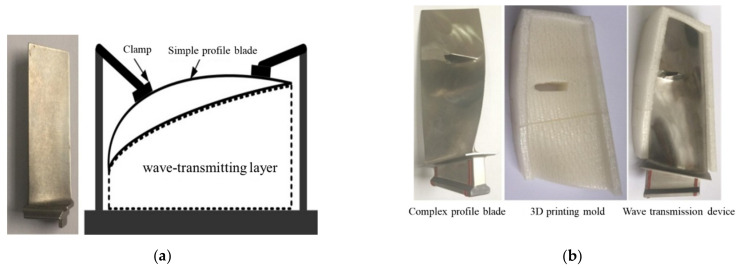
Two different materials are used for shock wave control. (**a**) “Hard” wave-transmitting layer; (**b**) “soft” wave-transmitting layer.

The first wave-transmitting method: using the “hard” wave-transmitting layer of the same material. For the simple profile blade, add a block of the same material and the same profile on the back of the blade. When the blade is fully attached to the block, use a clamp to fix it (Figure 17a), so as to ensure that the laser shock wave is smoothly transmitted from the back.

The wave-transmitting method uses blade material as the wave-transmitting material to ensure that the acoustic impedance of the wave-transmitting layer and the blade are completely matched. And the structure of the wave-transmitting layer is simple, but it is only suitable for simple-shaped blades. In addition, after a large area of laser peening, the blade will deform, leading to the wave-transmitting layer being unable to completely be attached to the back of the blade.

The second wave-transmitting method uses a “soft” wave-transmitting layer of compound materials. Through the mixing of different powder materials and solvents, a flexible wave-transmitting material similar to the acoustic impedance of the blade is formulated; 3D printing technology is used to create a mold with the same shape as the blade, and the wave-transmitting material is loaded into the mold. Finally, the blade is placed in the mold and compacted before the laser peening, as shown in Figure 17b.

The materials and density used in the preparation of the wave-transmitting materials are copper powder (2.6 g/cc), titanium powder (4.5 g/cc), nickel powder (8.8 g/cc), silver powder (10.5 g/cc), polyamide resin (0.88 g/cc), epoxy resin (0.98 g/cc), and acetone (0.785 g/cc). In order to make the density of the wave-transmitting material and the titanium alloy blade basically the same (Equation (4)), the preparation of the wave-transmitting material is as titanium powder: nickel powder: silver powder: polyamide resin: epoxy resin: acetone = 10:8:10:2:3:5:2 (volume ratio).

(4) $$\rho =\frac{\sum \rho_{i}V_{i}}{\sum V_{i}}=\rho_{Ti}$$

**Figure 18:**
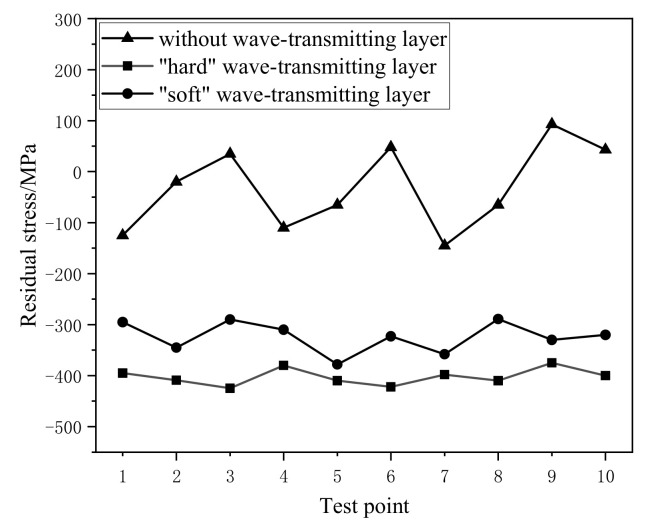
Surface residual stress under different wave transmission modes.

Figure 18 shows the residual stress profile on the surface under the same peening process (1064 nm/6 J/20 ns/3 mm, overlap rate 50%) and different wave transmission modes. The test points are a row of equidistant points on the surface of the specimen (D = 2 mm). The results show that there is a residual tensile stress area of about 100 MPa without the wave-transmitting layer, and the standard deviation of data is 72.71; it shows that the stress profile is uneven. After the wave-transmitting layer is applied, a large residual compressive stress is formed on the surface: the residual compressive stress of an average of 400 MPa is formed in the first method; the standard deviation is 15.44. While the residual compressive stress under the second method is about 310 MPa, the standard deviation is 28.26. The results show that the "hard" wave-transmitting layer can implant more residual compressive stress, and the profile of stress is more uniform. The main reason is that although the density of the wave-transmitting material in the second method is basically the same as titanium alloy, the elastic modulus is still different, and the acoustic impedance is not completely matched.

## 4. Conclusions

Aiming at the problem that the gradient residual compressive stress profile of laser peening thin blades cannot be formed, the finite element method is used to obtain the distribution law of the residual stress-strain profile. Compared with the infinite thickness plate model, the propagation law of shock wave and the dynamic response of material are analyzed. Response characteristics reveal the formation mechanism of the residual stress-strain profile. The reflection and coupling of the shock wave in the thin blade are suppressed through two wave transmission methods, and the effective control of the residual stress profile of the laser shock gradient of the thin blade is realized, and the experimental verification is carried out. The main conclusions are as follows:

1. Compared with the infinite thickness model, the equivalent plastic strain of the sheet after laser shock increases significantly in radial direction and depth, resulting in the formation of tensile residual stresses up to 410 MPa in the radial direction of the spot 0.5 mm and the depth of 0.3 mm. Although the residual compressive stress is formed out-side the radius of 0.5 mm, its value is quite small compared to the infinite thickness model, only 200 MPa.
2. Under the condition of the infinite-thickness model, the longitudinal wave attenuates exponentially and linearly in the elastic-plastic wave stage and the elastic wave stage, respectively. The shock wave will be reflected repeatedly in the sheet, and the property will change repeatedly between compression wave and extension wave. The reflected shock wave will be coupled with the subsequent shock wave to increase the pressure, and the peak pressure will be formed at the subsurface 0.2 mm.
3. Compared with the infinite thickness model, the transverse plastic strain of the sheet surface formed under the action of the first compression wave will be plastic deformation again under the action of the subsequent reflected tensile wave, so that the transverse plastic strain gradually decreases and the material is in the transverse compression state. This is the direct cause of the increase of equivalent plastic strain and the formation of residual tensile stress.
4. When using single-sided laser shock thin blades, using a "hard" wave-transmitting layer of the same material as the blade or a "soft" wave-transmitting layer configured with powder and solvent can effectively reduce the shock wave reflection intensity and improve the strengthening effect. And a better impedance matching effect is achieved under the condition of the "hard" wave-transmitting layer; compared with the "soft" wave-transmitting layer, the residual compressive stress is increased by about 100 MPa.

## Figures and Tables

**Table 1:** TC17 material parameters.

Parameters	Value
Density/(g·cm^−3^)	4.68
Elastic modulus/GPa	113
Poisson’s ratio	0.33
Elastic limit/GPa	2.9
A/MPa	1231
B/MPa	356.1751
*n*	0.2623
c	0.0197

## Data Availability

The data presented in this study are available upon request from the corresponding author.
